# Mild cognitive impairment prediction based on multi-stream convolutional neural networks

**DOI:** 10.1186/s12859-024-05911-6

**Published:** 2024-09-12

**Authors:** Chien-Cheng Lee, Hong-Han (Hank) Chau, Hsiao-Lun Wang, Yi-Fang Chuang, Yawgeng Chau

**Affiliations:** 1 Department of Electrical Engineering, Yuan Ze University, Taoyuan, 320 Taiwan; 2 Institute of Public Health, College of Medicine, National Yang Ming Chiao Tung University, Taipei, 112 Taiwan; 3 Department of Psychiatry, Far Eastern Memorial Hospital, New Taipei City, 220 Taiwan

**Keywords:** MCI, ResNet, CNN, Deep learning, Facial features

## Abstract

**Background:**

Mild cognitive impairment (MCI) is the transition stage between the cognitive decline expected in normal aging and more severe cognitive decline such as dementia. The early diagnosis of MCI plays an important role in human healthcare. Current methods of MCI detection include cognitive tests to screen for executive function impairments, possibly followed by neuroimaging tests. However, these methods are expensive and time-consuming. Several studies have demonstrated that MCI and dementia can be detected by machine learning technologies from different modality data. This study proposes a multi-stream convolutional neural network (MCNN) model to predict MCI from face videos.

**Results:**

The total effective data are 48 facial videos from 45 participants, including 35 videos from normal cognitive participants and 13 videos from MCI participants. The videos are divided into several segments. Then, the MCNN captures the latent facial spatial features and facial dynamic features of each segment and classifies the segment as MCI or normal. Finally, the aggregation stage produces the final detection results of the input video. We evaluate 27 MCNN model combinations including three ResNet architectures, three optimizers, and three activation functions. The experimental results showed that the ResNet-50 backbone with Swish activation function and Ranger optimizer produces the best results with an F1-score of 89% at the segment level. However, the ResNet-18 backbone with Swish and Ranger achieves the F1-score of 100% at the participant level.

**Conclusions:**

This study presents an efficient new method for predicting MCI from facial videos. Studies have shown that MCI can be detected from facial videos, and facial data can be used as a biomarker for MCI. This approach is very promising for developing accurate models for screening MCI through facial data. It demonstrates that automated, non-invasive, and inexpensive MCI screening methods are feasible and do not require highly subjective paper-and-pencil questionnaires. Evaluation of 27 model combinations also found that ResNet-50 with Swish is more stable for different optimizers. Such results provide directions for hyperparameter tuning to further improve MCI predictions.

## Background

Mild Cognitive Impairment (MCI) is a transition from normal aging to dementia, and about 50% of patients with MCI progress to Alzheimer's disease (AD) within 5 years [1]. AD is a neurodegenerative disorder characterized by cognitive decline with loss of memory. Once MCI enters the dementia stage, caring for these patients becomes complicated and costly. Early identifying patients with MCI and timely applying treatment can delay the progress of the MCI to AD [2]. However, the symptom of MCI is often neglected due to inconvenient, expensive, and/or time-consuming methods for its early detection. Therefore, the early diagnosis of MCI plays an important role in human healthcare.

Current methods of MCI detection include cognitive tests to screen for executive function impairments, possibly followed by neuroimaging tests. Two common cognitive screening tests for MCI are the Mini-Mental State Examination (MMSE) [3] and the Montreal Cognitive Assessment (MoCA) [4]. Cognitive tests are not completely objective and may be influenced by the conducting physician or the patient's age and educational background [5]. Furthermore, neuroimaging methods are expensive and time-consuming, making them unsuitable for screening large populations. These neuroimaging techniques typically include positron emission tomography (PET), single-positron emission computed tomography (SPECT), and functional magnetic resonance imaging (fMRI). Consequently, a non-invasive, cost-effective, and easy-to-use screening method is critical for detecting MCI.

Several studies have demonstrated that MCI and dementia can be detected by machine learning technologies from different modality data, such as naturalistic driving data [6], speech data [7–9], and facial data [10]. Traditional machine learning consists of two steps: feature extraction and classification. These two steps are closely related. If feature extraction produces bad results, classification has to work hard for better performance. The intrinsic properties of the modality often affect feature extraction, and some latent features are difficult to be extracted and tracked by humans.

The use of facial data to detect MCI and dementia has attracted the attention of many researchers because of its easy availability. Most of them use static facial images to extract facial expressions and features such as action units [11], eye gaze [12], and lip activity [13]. However, static images only represent spatial features and lack temporal variation. Changes in faces over time should contain more information than static images. In other words, more complete facial features include not only spatial features, but also motion features when people respond to certain questions. We believe that the combination of spatial and motion features can provide better facial representation and improve MCI detection. Thus, capturing and modeling the spatial and motion features are essential for MCI detection through facial data.

Convolutional neural networks (CNNs) are able to automatically extract features from large amounts of data, rather than traditional machine learning using handcrafted features. The success of CNNs in object classification has recently prompted researchers to leverage their feature learning capabilities to solve many computer vision problems through variants of CNNs. Among them, a two-stream architecture is the basis of most current models for behavior recognition and emotion recognition problems [14–17]. The architecture contains two CNNs: a spatial network that processes a static image, and a temporal network that processes motion information, most commonly represented by the optical flow. It can simultaneously learn spatial and motion features, especially low-level short-term facial motion features in the temporal stream.

Inspired by this, this study proposes a multi-stream CNN (MCNN) model to predict MCI from face videos. A face video is divided into several segments. For each segment, MCNN extracts and learns facial features representing spatial features from RGB image frames and motion features from motion vector sequences. In this way, the latent static facial features and smaller micro-motion features can be captured. Then, the fusion stage combines the spatial and temporal features to form feature vectors, and the classification stage predicts the MCI detection results for each segment. Finally, the aggregation stage produces the final detection results of the input video. We evaluate the performance of our method on 48 videos from 45 participants. The test results show that the proposed method achieves the best results at segment level and participant level with F1 scores of 89% and 100%, respectively. It shows that an automatic, non-invasive, and inexpensive MCI screening method from facial videos is feasible, without the requirement for highly subjective paper-and-pencil questionnaires. Our key contributions are as follows:

- We combine spatial and motion features to provide better facial representation and improve MCI detection.
- We demonstrate the effectiveness of the MCNN model based on spatial and motion features to detect MCI.
- We investigate the impact of different optimizers and activation functions on the performance of different deep residual network (ResNet) [18] architectures and provide direction for hyperparameter tuning.

## Literature review

MCI detection is an active topic of research. Some biomarkers are commonly used to detect MCI, such as cognitive tests, electroencephalogram (EEG), speech, facial images, and neuroimaging tests. De Jager et al. [19] evaluated whether the computerized cognitive test battery, CogState, was as sensitive to MCI as two well-validated ‘paper-and-pencil’ tests, the Hopkins Verbal Learning Test (HVLT) [20] and the MMSE. Biomarkers recorded from EEG such as event related potentials (ERPs) have been used extensively in observing electrophysiological activities in MCI and AD populations. White et al. [21] combined EEG biomarkers into a multidimensional feature space allowed for differentiation between healthy and MCI participants based on their respective MoCA scores. Rutkowski et al. [22] proposed a machine learning-based MCI detection using behavioral responses. The classifier input features included emotional valence and arousal recognition responses in older adults, as well as reaction times.

Over the past decade, several results have been published in the particular domain of speech-based cognitive impairment (CI) detection [7, 8]. Speech reveals multidimensional information about the speaker (e.g., age, gender, sociolinguistic characteristics, physiological condition) and can function as a fingerprint that identifies patients with MCI from healthy controls. Themistocleous et al. [9] investigated whether voice quality and speech fluency distinguish patients with MCI from healthy individuals to improve diagnosis of patients with MCI. Their findings provide objective measures of voice quality that can distinguish MCI patients from healthy controls. At the same time, they point to the importance of phonation and speech fluency as diagnostic measures. Remote-automated cognitive impairment monitoring has the potential to facilitate the care of the elderly with mobility restrictions. Yu et al. [23] proposed a speech-based CI detection from remotely-collected cognitive test audio to improve remote cognitive health monitoring.

One of the most important and useful biomarkers is neuroimaging test. In recent work, deep learning techniques have been widely used for medical image analysis. Yang et al. [24] proposed a neuroimaging method to identify MCI using a deep learning method and functional near-infrared spectroscopy (fNIRS). Hedayati et al. [25] used a set of pretrained autoencoder-based feature extraction modules to generate image features from 3D input images, and then used a CNN to diagnose AD. Resting-state functional magnetic resonance imaging (rs-fMRI) using blood-oxygen-level-dependent (BOLD) signals as neurophysiological indicators has been widely applied to identify neurodegenerative diseases, especially for MCI and AD [26]. Current studies focus on using dynamic functional connectivity (dFC) to identify brain disorders [27, 28]. Li et al. [29] developed a novel adaptive dFC model, aided by a deep spatial–temporal feature fusion method for MCI identification.

More recent approaches have aimed to use computer vision techniques to detect MCI/dementia through facial data [30]. Tanaka et al. [31] proposed a method to automatically detect dementia from a human face. They identified various contributing features, such as action units, eye gaze, and lip activity. Wang et al. [32] compared different deep learning methods for assessing facial dynamics such as talking, singing, neutral and smiling in AD-patients. These methods include 3D CNNs, two-stream CNNs, as well as improved dense trajectories. The two-stream CNNs in combination with ResNet-152 obtains the best performance on their dataset. The artificial intelligence-based facial expression recognition systems are also used to predict neuropsychiatric symptoms of persons with dementia and screen people with cognitive impairment [10, 33].

## Materials and methods

### Data collection

**Fig. 1:**
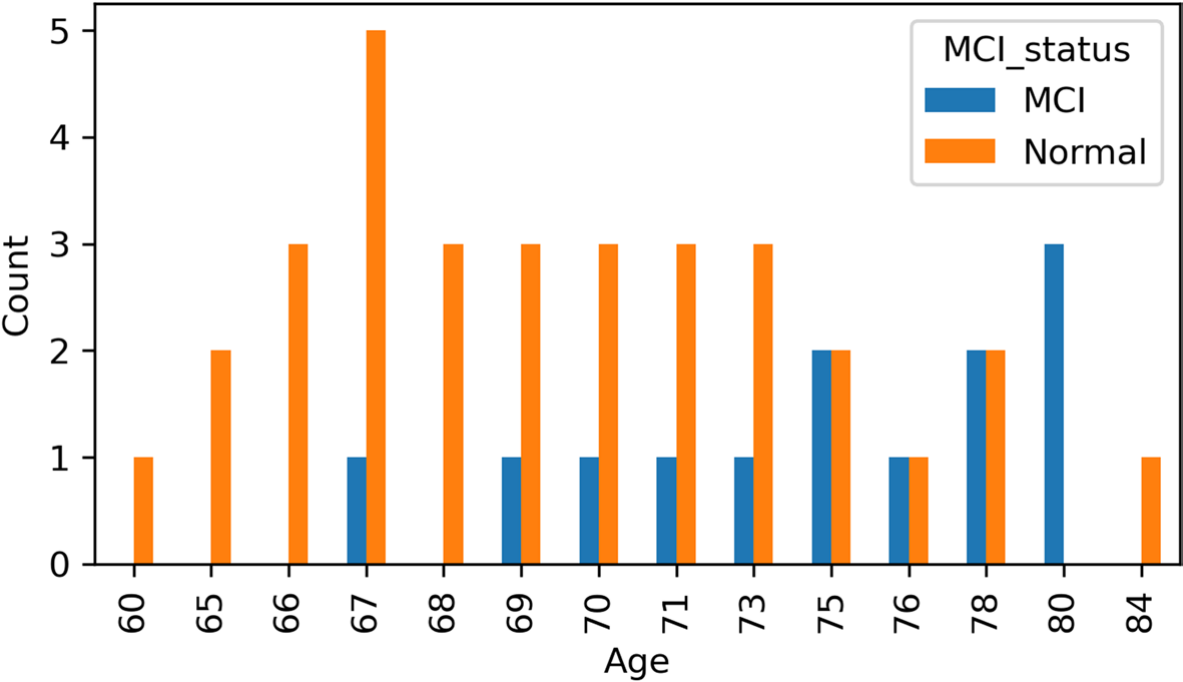
Age distribution of participants

All participants gave their informed consent for inclusion before they participated in the study. The study was conducted in accordance with the Declaration of Helsinki, and the protocol was approved by the Far Eastern Memorial Hospital Research Ethics Committee (105147-F) and the Institutional Review Board of the National Yang-Ming University (YM108110E). There are 45 participants in this study, 32 are cognitively normal (median age 69 years, IQR 67–73 years, 9 males, 23 females) and 13 are diagnosed with MCI (median age 75 years, IQR 71–78 years, 6 males, 7 females). Table 1 and Fig. 1 show the gender and age distribution of the participants. In order to collect realistic and reasonable data from participants without stress or embarrassment, participants recorded videos while participating in the MMSE.

**Table 1:** Gender distribution of participants

	Female	Male	Total
Normal	23	9	32
MCI	7	6	13
Total	30	15	45

The total effective data are 48 facial videos from 45 participants, including 35 videos from normal cognitive participants and 13 videos from MCI participants. Several types of resolutions are used in the original videos, such as 1920 × 1080, 1280 × 720, and 640 × 480. The video frame rate is 29.97 frames per second (fps). Video lengths range from 3 to 30 min, and the average length is 14.5 min. To reduce the spatial and temporal redundancy before processing, the frame resolution was also resized to 640 × 480, and the video frame rate was down-sampled to 5 fps.

### MCI prediction model

**Fig. 2:**
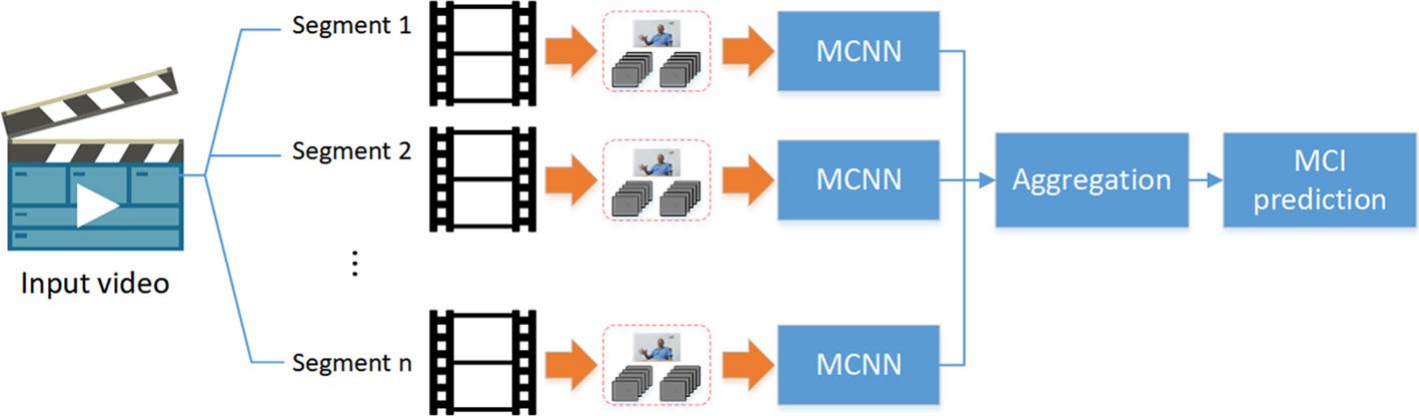
Overview of the MCI prediction model

We proposed an MCI prediction model based on MCNNs to predict whether a participant video is MCI, as shown in Fig. 2. First, a participant video is divided into several segments. Then, we generate spatial and motion data streams as input to MCNN for each segment. MCNN captures latent spatial and motion features from the data streams to extract facial representations during MMSE testing, and classifies segments as MCI or normal based on these facial features. Finally, the aggregation stage produces the final detection results of the input video.

We randomly sample a frame from each segment to generate the spatial data stream. The frame is an RGB image that contains the participant's face, which can be used to represent static facial spatial information. During the MMSE test, the participants' facial responses are also important. To capture the facial dynamics, the motion data stream is generated from segment frames using optical flow techniques [34]. Optical flow is used in computer vision to obtain the motion field on individual pixel basis between two image frames. It is widely used in a variety of biomedical applications for tracking changes over time [35, 36]. The stacked optical flow fields with x and y directions are calculated to represent facial motion information. In this study, we choose the TVL1 optical flow algorithm [37] implemented by OpenCV with CUDA.

**Fig. 3:**
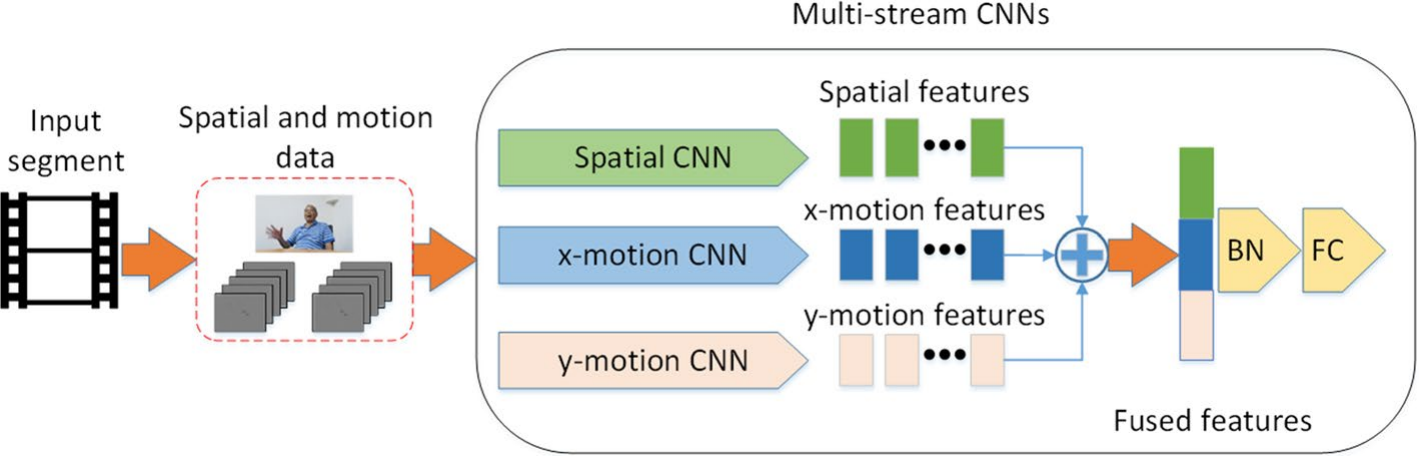
Architecture of MCNN model

Inspired by two-stream CNNs [14, 17], our MCNN mainly consists of three CNNs, a fusion mechanism, and a fully connected layer as a classifier, as shown in Fig. 3. The three CNNS are spatial CNN, x-motion CNN, and y-motion CNN. It receives the spatial and x, y motion streams from a segment as inputs, and uses the three CNNs to extract facial spatial and motion features. The spatial features, x-motion features, and y-motion features are then concatenated to form a one-dimensional vector. Finally, the fused feature vector is classified as MCI or normal through a batch normalization (BN) layer and a fully connected (FC) layer.

MCNN acts as a segment classifier in the MCI prediction model. For each segment, each MCNN classifier produces a unique decision regarding the identity of the segment. Finally, a majority voting scheme [38] is used as an aggregation of classifiers. In aggregating the decisions of the *n* MCNN classifiers, the input video is assigned to the MCI class when at least *k* MCNN classifiers agree, where

1 $$k = \left\{ {\begin{array}{*{20}c} {\frac{n}{2} + 1} & {\quad {\text{if }}n{\text{ is even}}} \\ {\frac{n + 1}{2}} & {\quad {\text{if }}n{\text{ is odd}}.} \\ \end{array} } \right.$$

The MCNN is a general and flexible model at the segment level. Several modern CNN models can be used as the backbone of MCNN. In order to train our MCNN to perform optimally, we choose ResNet as the backbone, after considering its balance between accuracy and efficiency. Meanwhile, most CNN models provide pre-trained models based on working with the public ImageNet dataset [39]. The transfer learning allowed building a high-quality classification model for new data, based on a small amount of newly labeled data. Therefore, we used the transfer learning to fine-tune the pre-trained CNNs to expedite training and to increase accuracy. In the transfer learning, we unfreeze and train the last convolutional block of the pre-trained model, as well as the top-layer classifier (FC layer). In this way, we retain the generic features learned from the ImageNet dataset, while learning domain knowledge from the facial video data.

### MCNN exploration

Although MCNN captures and learns spatial and motion features to predict MCI from video segments, the accuracy of MCI prediction also depends on the model architecture. Therefore, exploring different types of model architectures is necessary to devise a robust solution. To further attempt to improve model accuracy, we explored and compared the following model settings and their combinations:

1. ResNets with different numbers of layers, namely ResNet-18, ResNet-34, and ResNet-50.
2. ReLU, Swish, and Mish activation functions [40, 41] in ResNets.
3. SGD, Adam, and Ranger [42] optimizers in model training.

The activation function plays an important role in neural network training. In the early era of the neural network, sigmoid function was the most used activation function in neural networks. However, its small derivative may cause the vanishing gradient problem, so ReLU is more suitable and widely used in deep learning because it has a derivative of one for every positive input. Nevertheless, if the weights in the network always lead to negative inputs into a ReLU neuron, the neuron output is zero and it is dead. This phenomenon is called the dying ReLU problem. Several variants of ReLU have been proposed that perform as well or better than ReLU. Unfortunately, none of them have achieved the same popularity as ReLU due to its simplicity [43].

Swish is a smooth non-monotonic activation function, similar to ReLU. The Swish activation function is defined as follows [40]:

2 $${\text{Swish}}\left( x \right) = \frac{x}{{1 + e^{ - x} }}$$

The simplicity of Swish and its similarity to ReLU means that replacing ReLUs in any network is just a simple one line code change. Even this simple, empirical performance shows that Swish consistently outperforms ReLU and other activation functions. Mish is a new activation function with similar shape and properties to Swish, defined as follows [41]:

3 $${\text{Mish}}\left( x \right) = x{\text{tanh}}\left( {{\text{log}}\left( {1 + e^{x} } \right)} \right)$$

**Fig. 4:**
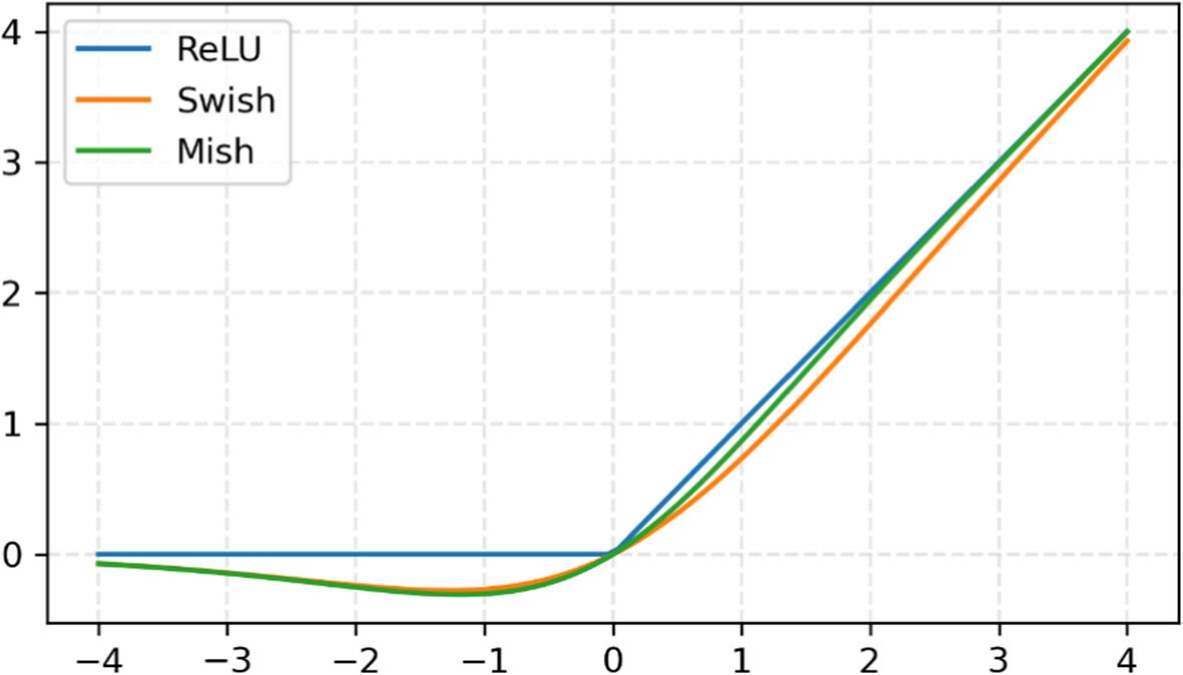
Graphs of ReLU, Swish, and Mish activation functions

The graphs of ReLU, Swish, and Mish are shown in Fig. 4. As shown in Fig. 4, the main difference is the concave part of the function. Mish keeps improving ReLU and Swish at the cost of more computation. In this study, we compare the performance of ReLU, Swish, and Mish in ResNets to find the best model architecture.

Optimizers are critical to the performances of neural networks. While a large number of optimizers are proposed, most of these publications provide incremental improvements to existing algorithms. We adopted the current state-of-the-art optimizer Ranger to improve model training. The Ranger optimizer combines two emerging works from RAdam and Lookahead to build a set of optimizers for deep learning. RAdam uses a dynamic rectifier to adjust Adam's adaptive momentum based on variance and effectively provides an automatic warm-up mechanism. LookAhead can provide strong and stable breakthroughs throughout the training process. Therefore, the inventor of the Ranger claim that combining the two can achieve higher accuracy. This study also compares the performance of SGD, Adam, and Ranger optimizers in model training.

### Generating training and test segments

In the MCI prediction mode, only the MCNN needs to be trained. Therefore, we divide each participant video into several segments to generate the training and test segments. In this study, considering the video length, and because the number of MCI videos is smaller than the number of normal videos, we evenly extract 200 segments from MCI videos, and 100 segments from normal videos to balance MCI and normal classes. In the end, a total of 5154 segments are extracted from 48 videos, some of which are too short to extract enough segments. Each segment contains 10 frames, and a segment is considered a processing unit of the MCNN.

To generate the training and validation segments, we need to split all segments into training and validation sets. However, we cannot directly split the segments because they may come from the same participant. During the training process, the validation data should not be visible. If the training and validation segments come from the same participant, it means that data has been learned during the training. Therefore, this study uses a two-stage approach to generating the training and validation segments.

**Fig. 5:**
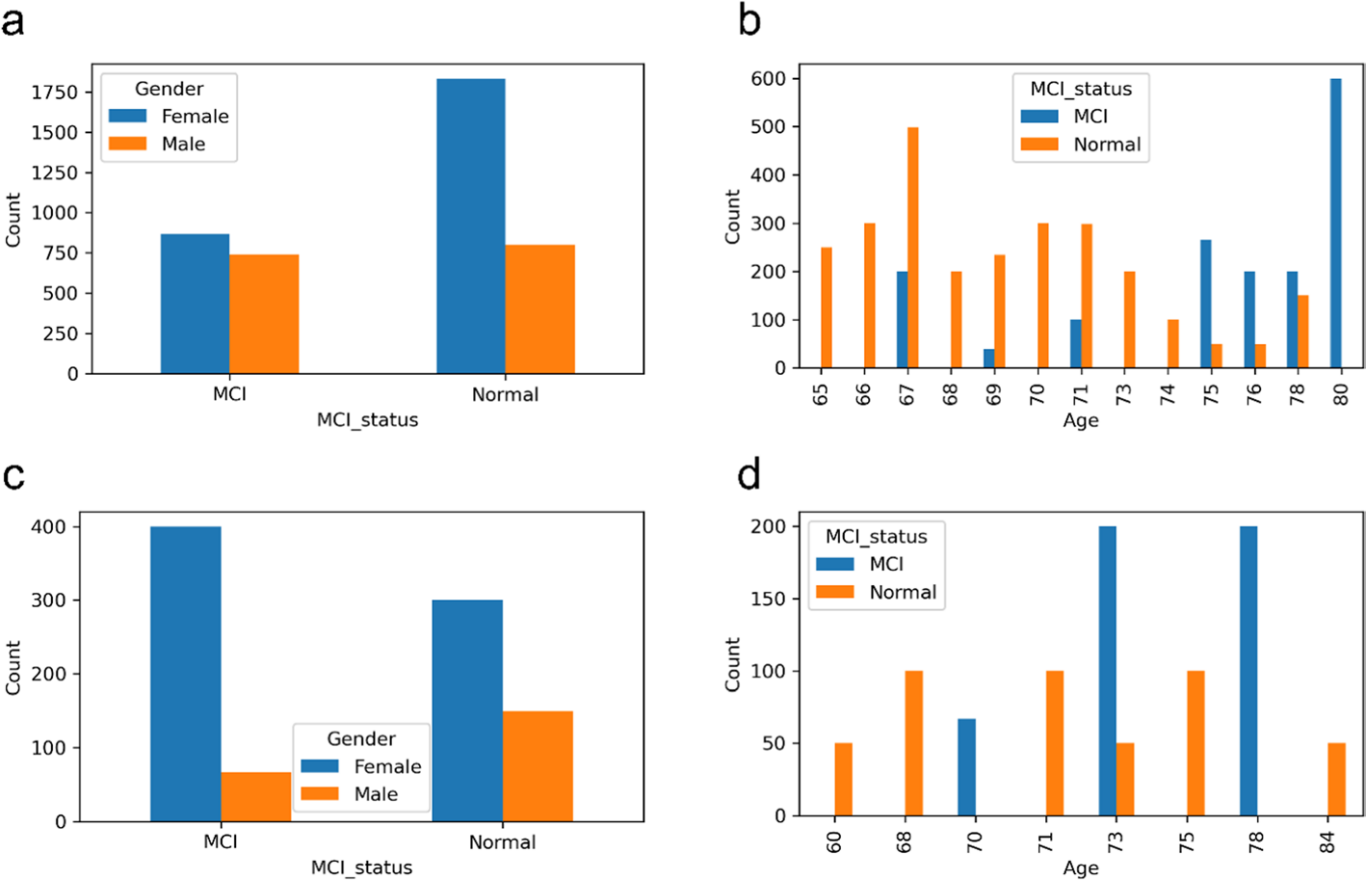
The gender and age distributions of the segmented training set and validation set. **a** Gender distribution of the segmented training set. **b** Age distribution of the segmented training set. **c** Gender distribution of the segmented validation set. **d** Age distribution of the segmented validation set

First, all participants are randomly grouped into training and validation groups in a ratio of approximately 8:2. We use the stratified K-fold cross-validation implemented by scikit-learn library [44] to split the participants into groups with roughly the same proportions of classes in the original data. Then, after the participant grouping, all segments are divided into training and validation sets according to their corresponding participant IDs in the training or validation groups. Table 2 shows the numbers of training and validation sets. There are 4237 segments (36 participants, 39 videos) in the training set and 917 segments (9 participants, 9 videos) in the validation set. MCI segments are marked as positive and normal segments are marked as negative. Because there are not many video data, we do not have a separate test set. The verification set will be used in the model testing phase to evaluate the model testing performance. The gender and age distributions of the segmented training set and validation set are shown in Fig. 5.

**Table 2:** Numbers of training and validation sets

	Training set			Validation set		
	Participants	Videos	Segments	Participants	Videos	Segments
Normal	26	29	2631	6	6	450
MCI	10	10	1606	3	3	467
Total	36	39	4237	9	9	917

## Experimental results

### Implementation details and settings

We conducted all experiments on a computer with Intel(R) Xeon Silver 4110 CPU and NVIDIA Tesla V100-32 GB GPU. The PyTorch deep learning framework is used to implement our model. We initialize the spatial CNN weights using the pre-trained model from ImageNet. At the same time, we compute the average weights of the RGB channels of the pre-trained model and initialize the weights of the motion CNNs with these average weights. Regarding data augmentation, we use the techniques of multi-scale cropping and random horizontal flipping.

As mentioned earlier, to further improve model accuracy, we explored and compared the model settings and their combinations, including different architectures, activation functions, and optimizers in ResNet training. Among them, we uniformly set the initial learning rate to 0.001, the batch size to 25, and the training epoch to 30, respectively. An exponential learning rate decay is also used in model training. The best mode during the training will be stored and used for testing.

Four metrics are employed to measure the quantitative impact of prediction results between different model settings. These metrics are precision, recall, accuracy, and F1-score, as follows:

4 $$Accuracy = \frac{{{\text{No}}.\;{\text{of}}\;{\text{Correct}}\;{\text{Predictions}}}}{{{\text{No}}.\;{\text{of}}\;{\text{Total}}\;{\text{Predictions}}}} = \frac{TP + TN}{{TP + FP + FN + TN}}$$

5 $$Recall = \frac{{{\text{No}}.\;{\text{of}}\;{\text{Correctly}}\;{\text{Predicted}}\;{\text{Positive}}\;{\text{Instances}}}}{{{\text{No}}.\;{\text{of}}\;{\text{Total}}\;{\text{Positive}}\;{\text{Instances}}\;{\text{in}}\;{\text{Dataset}}}} = \frac{TP}{{TP + FN}}$$

6 $$Precision = \frac{{{\text{No}}.\;{\text{of}}\;{\text{Correctly}}\;{\text{Predicted}}\;{\text{Positive}}\;{\text{Instances}}}}{{{\text{No}}.\;{\text{of}}\;{\text{Total}}\;{\text{Positive}}\;{\text{Predictions}}}} = \frac{TP}{{TP + FP}}$$

7 $$F1 Score = \frac{{2 \times {\text{Precision}} \times {\text{Recall}}}}{{{\text{Precision}} + {\text{Recall}}}}$$

where TP, TN, FP, and FN represent the true positive, true negative, false positive, and false negative, respectively. Accuracy is defined as the ratio of true positives and true negatives to all instances. In other words, it is the fraction of correct predictions. Precision quantifies the number of positive class (i.e., MCI) predictions that actually belong to the positive class. Recall quantifies the number of positive class predictions made out of all positive instances in the dataset. There is a trade-off between precision and recall according to their definitions. F1-score provides a way to combine both precision and recall into a single measure that captures both properties. Precision, recall, and F1-score provide better insights into predictions than accuracy.

### Model architecture evaluation

To better build an accurate prediction model suitable for our dataset, we use ResNet-18, ResNet-34, and ResNet-50 as the MCNN backbones to build MCI prediction models, respectively. In each ResNet, we further analyze the performance using three different optimizers (SGD, Adam and Ranger) and three different activation functions (ReLU, Swish and Mish). Figure 5 shows the F1-score of the test results for different model combinations at the segment level. The three columns of Fig. 5 show the test results of MCNN using ReLU, Swish, and Mish activation functions in ResNets. The three rows of Fig. 5 show the test results of the MCNN trained by Adam, Ranger, and SGD optimizers.

**Fig. 6:**
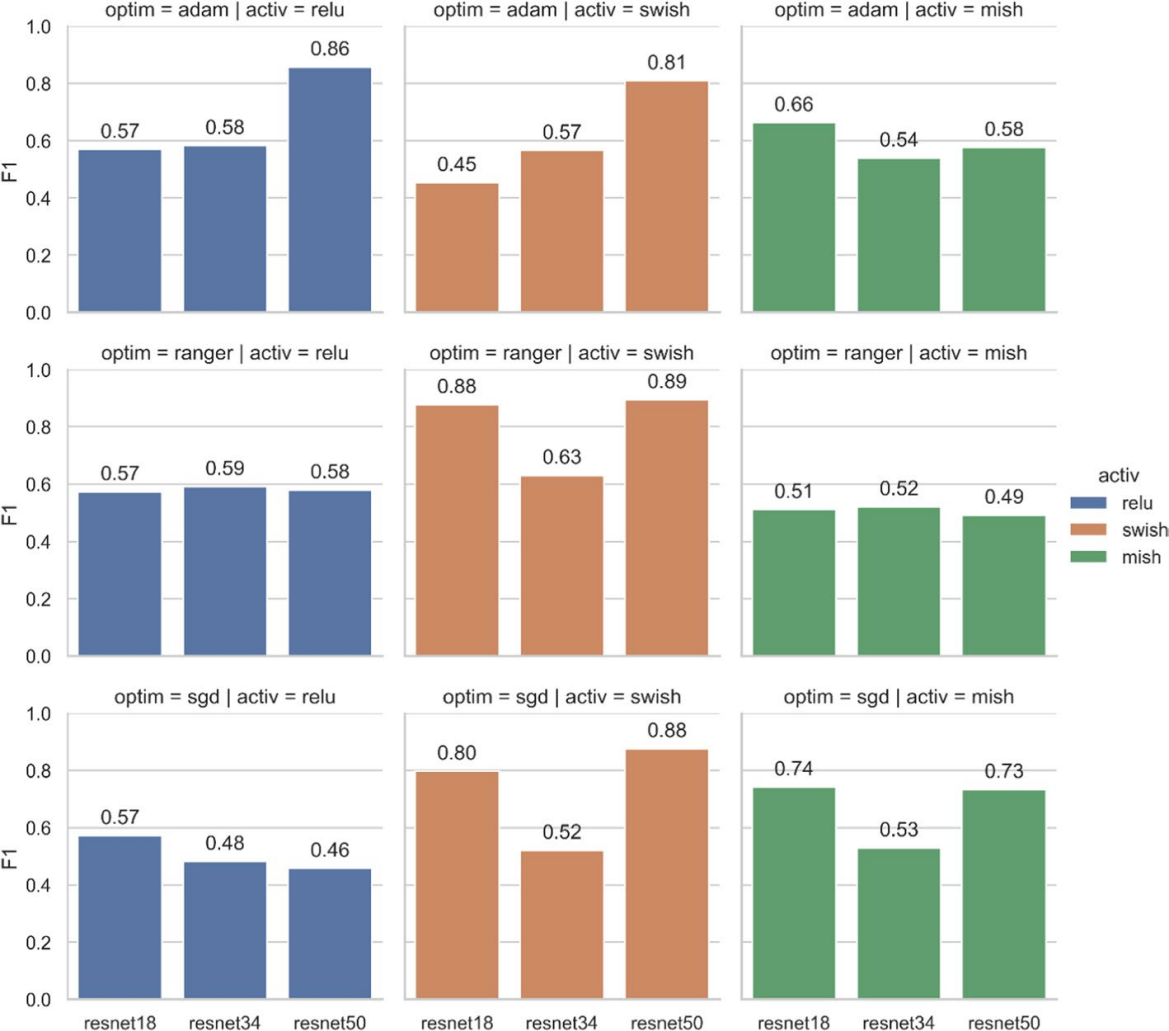
F1-score of the test results for different model combinations at the segment level

Figure 6 shows that the activation functions and optimizers greatly affect the performance. The model using ReLU activation function and SGD optimizer achieves the worst results, as shown in the lower left of Fig. 5. ResNet-50 backbone with Swish activation function and Ranger optimizer produces the best results with an F1 score of 0.89. On average, the activation functions of ReLU and Mish perform poorly (F1-score < 0.75), but ResNet-50 using ReLU and Adam yields better results with an F1-score of 0.86. Surprisingly, Mish, as a novel activation function, does not perform well in our dataset.

**Fig. 7:**
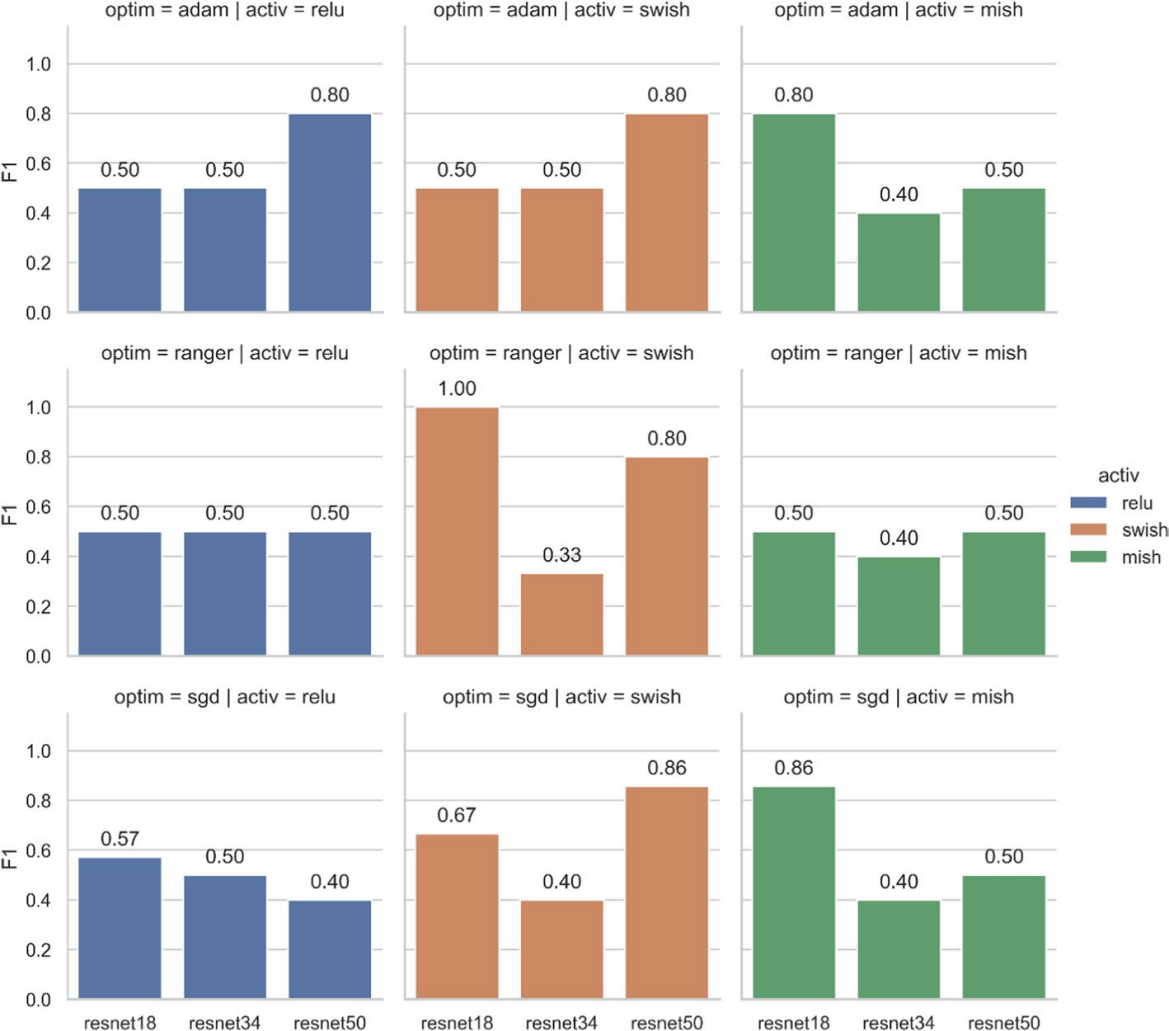
F1-score of the test results for different model combinations at the participant level

Figure 7 shows MCI prediction results at the participant level. We use a majority voting scheme to aggregate segments of the same participant and predict the participant as MCI or normal. The best result is the combination of ResNet-18 backbone with Swish activation function and Ranger optimizer, with an F1-score of 1. The next best combinations are ResNet-50 backbone with Swish and SGD, and ResNet-18 backbone with Mish and SGD. Both achieve an F1-score of 0.86. It is worth mentioning that we used the same learning rate for all three optimizers in our study. However, different optimizers may require different learning rates. Models may reach good or very poor accuracy for some ranges of the learning rate. Although ResNet-18 with Swish and Ranger achieves the best results at the participant level, ResNet-50 with Swish is more stable for different optimizers. This finding could provide direction for hyperparameter tuning.

**Fig. 8:**
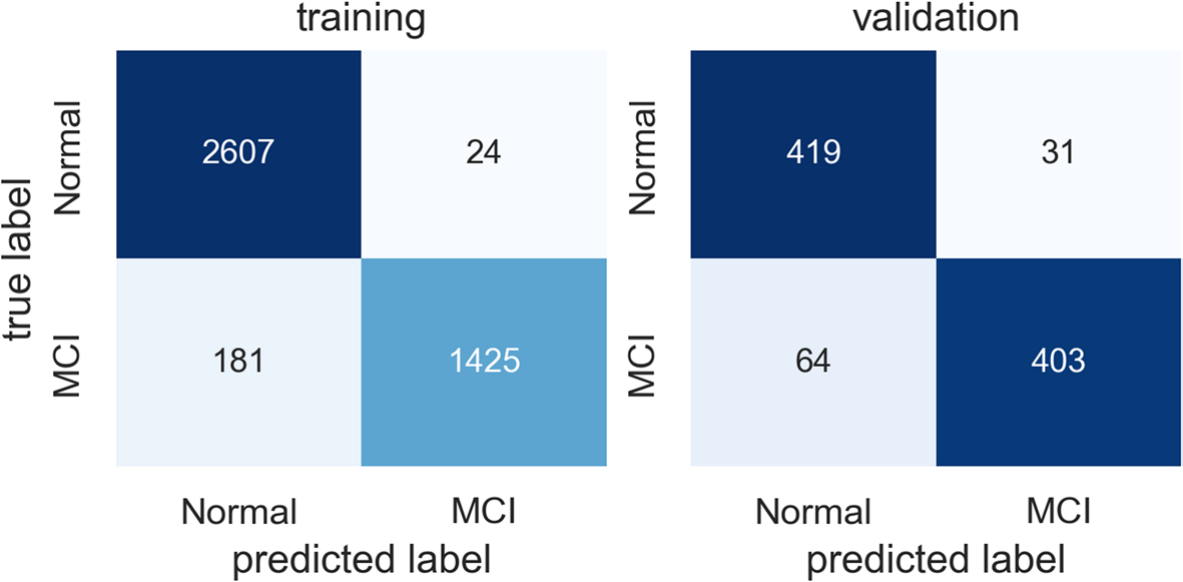
Confusion matrix for MCI prediction at segment level

**Fig. 9:**
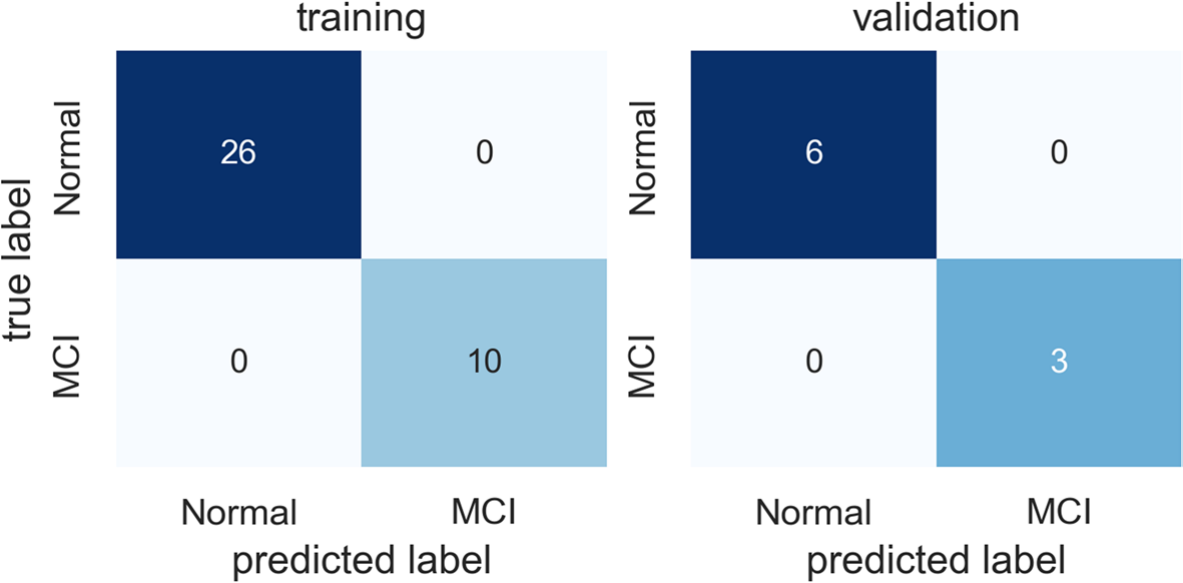
Confusion matrix for MCI prediction at participant level

Table 3 summarizes the precision, recall, accuracy, and F1-score results of the models using Swish and Ranger. While the results show that the ResNet-18 backbone with Swish and Ranger achieves the F1-score of 100% at the participant level, the same combination model achieves only the F1-score of 88% at the segmentation level. This is because we use the majority voting scheme in the participant MCI prediction, and the decision depends on the distribution of misclassified segments between participants. Figure 8 shows the confusion matrix for MCI prediction at segment level using ResNet-50 backbone with Swish and Ranger. Figure 9 shows the confusion matrix for MCI prediction at participant level using ResNet-18 backbone with Swish and Ranger. In Fig. 8, the misclassification rate of the MCI segments is higher than that of the normal segments. This may be due to data imbalance. The MCI data is less than healthy data in our dataset.

**Table 3:** Test results of MCI prediction models using Swish and Ranger

Level	Precision	Recall	Accuracy	F1	Backbone
Segment	0.86	0.90	0.87	0.88	Resnet18
Segment	0.61	0.65	0.61	0.63	Resnet34
Segment	0.93	0.86	0.90	0.89	Resnet50
Participant	1.00	1.00	1.00	1.00	Resnet18
Participant	0.33	0.33	0.56	0.33	Resnet34
Participant	1.00	0.67	0.89	0.80	Resnet50

## Discussion

MCI prediction from facial videos is a challenge. In this study, we propose a MCNN-based MCI prediction method. We evaluate ResNet-18, ResNet-34, and ResNet-50 as MCNN backbone networks, which combine three different activation functions (ReLU, Swish, and Mish) and three different optimizers (SGD, Adam, and Ranger), yielding 27 models. Our results show that the activation functions and optimizers greatly affect the performance. In participant-level evaluations, the results show that the ResNet-18 backbone with Swish and Ranger achieves the F1-score of 100%. In segment-level evaluations, the ResNet-50 backbone with Swish and Ranger produces the best results with an F1-score of 89%. Experiments show that ResNet-50 with Swish is more stable for different optimizers. This finding could provide direction for hyperparameter tuning.

Although our model has demonstrated good prediction performance at the participant level, there is still room for improvement in the MCNN. Here are a few areas for refinement:

*Increasing the dataset* Particularly for MCI cases, expanding the dataset is crucial. Deep learning models require large amounts of data to effectively train their parameters. To address the current limitations, we divided the video data into hundreds of segments to augment the dataset. However, having more participant data overall would significantly benefit the training process.

*Improving video quality* The quality of the videos varies significantly. Several videos had to be excluded due to poor quality, such as bad camera angles, improper distances, face mask occlusions, and other distracting foreground and background objects. Ensuring consistent and high-quality video recordings will enhance the reliability of the data.

*Analyzing video segments* In this study, we used the entire video recorded during the MMSE test. However, participants exhibit different states (e.g., listening, thinking, responding) at various times. Conducting a more detailed analysis of these distinct states could further improve the model's prediction performance.

## Conclusions

The MCNN effectively captures latent facial spatial features and dynamic movements from facial videos. By leveraging MCNN, we can obtain robust facial representations without relying on the handcrafted features typically used in traditional machine learning methods. Research indicates that MCI can be detected through facial videos, positioning facial data as a potential biomarker for MCI. This approach holds great promise for developing accurate models to screen for MCI using facial data. It underscores the feasibility of automated, non-invasive, and cost-effective MCI screening methods that do not depend on highly subjective paper-and-pencil questionnaires. Additionally, this approach could be extended to detect similar symptoms, such as the behavioral and psychological symptoms of dementia (BPSD) in individuals with dementia.

## Data Availability

The datasets generated and/or analyzed during the current study are not publicly available due to participant privacy but are available from the corresponding author on reasonable request.

## Abbreviations

MCI: Mild cognitive impairment
MCNN: Multi-stream convolutional neural network
CNNs: Convolutional neural networks
AD: Alzheimer's disease
MMSE: Mini-Mental State Examination
MoCA: Montreal Cognitive Assessment
PET: Positron emission tomography
SPECT: Single-positron emission computed tomography
fMRI: Functional magnetic resonance imaging
EEG: Electroencephalogram
HVLT: Hopkins Verbal Learning Test
ERPs: Event related potentials
CI: Cognitive impairment
fNIRS: Functional near-infrared spectroscopy
rs-fMRI: Resting-state functional magnetic resonance imaging
BOLD: Blood-oxygen-level-dependent
dFC: Dynamic functional connectivity
BN: Batch normalization
FC: Fully connected
ResNet: Deep residual network
BPSD: Behavioural and psychological symptoms of dementia
PwD: People with dementia
